# Phosphate-Solubilizing Bacterium *Acinetobacter pittii* gp-1 Affects Rhizosphere Bacterial Community to Alleviate Soil Phosphorus Limitation for Growth of Soybean (*Glycine max*)

**DOI:** 10.3389/fmicb.2021.737116

**Published:** 2021-09-24

**Authors:** Donglan He, Wenjie Wan

**Affiliations:** ^1^College of Life Science, South-Central University for Nationalities, Wuhan, China; ^2^Key Laboratory of Aquatic Botany and Watershed Ecology Wuhan Botanical Garden, Chinese Academy of Sciences, Wuhan, China; ^3^Center of the Plant Ecology, Core Botanical Gardens, Chinese Academy of Sciences, Wuhan, China; ^4^State Key Laboratory of Agricultural Microbiology, Huazhong Agricultural University, Wuhan, China

**Keywords:** phosphorus-solubilizing bacteria, P-cycling-related gene, rhizosphere bacterial community, functional profiling, vegetation properties

## Abstract

Phosphorus (P) availability is a major restriction to crop production, and phosphate-solubilizing bacteria (PSBs) in soils are responsible for P turnover. However, it remains unknown whether the application of PSB can facilitate both inorganic and organic P transformation and enhance function of plant rhizosphere bacteria. In this study, we applied Illumina MiSeq sequencing, plate-colony counting, quantitative PCR, and multiple ecological analyses. We found that the inoculation of PSB *Acinetobacter pittii* gp-1 significantly promoted the growth of soybean represented by better vegetation properties (e.g., plant height and root P) and increased activities of phosphatase (4.20–9.72 μg/g/h) and phytase (0.69–1.53 μmol/g/day) as well as content of indole acetic acid (5.80–40.35 μg/g/h). Additionally, the application of strain *A. pittii* gp-1 significantly increased abundances of both inorganic and organic P-cycling-related genes (i.e., *phoD*, *bpp*, *gcd*, and *pstS*). More importantly, the application of *A. pittii* gp-1 could increase the function represented by P-cycling-related enzymes (e.g., phosphotransferase) of rhizosphere bacterial community based on functional profiling. To our knowledge, this is the first report that the application of PSB *A. pittii* promotes inorganic and organic P utilization and increases the function of rhizosphere bacterial community. Therefore, the PSB *A. pittii* gp-1 could be a good candidate for the promotion of soybean growth.

## Introduction

Enhancing the yield of farmland is the most important agricultural issue (Mehrabi and Ramankutty, 2019). P is an essential element for growth and development of plants and, thus, is of significance to the production of fiber and food crops (Hansen et al., 2020; Wan et al., 2020a). At present, the major and wide input of P to farmland is non-renewable P fertilizer, which is often applied beyond the demand of crops due to soil P fixation to metal ions (Neal et al., 2017; Ye et al., 2017). The accumulation of P in soil could lead to the waste of resources and potential environmental risks (e.g., soil compaction and water eutrophication) (Hu et al., 2018). Rational fertilization and improving utilization efficiency of P fertilizer are important agricultural problems.

The transformation of plant-unavailable P (e.g., Ca_3_(PO_4_)_2_, phytate, phospholipid, and nucleic acid) to plant-available P (e.g., H_2_PO_4_^–^ and HPO_4_^2–^ ions) needs the participation of P-solubilizing microorganisms (Yu et al., 2011; Wan et al., 2020b). PSBs are responsible for the solubilization of inorganic P and mineralization of organic P (Oliveira et al., 2009; Liu et al., 2014). Phospholipids and phytate are significant organic P pools in soils, which can be hydrolyzed by phosphatase and phytase, respectively (Lim et al., 2007; Maougal et al., 2014; Wei et al., 2019). The inorganic P can be solubilized by small molecular organic acids (e.g., gluconic acid and citric acid), and the formation of small molecular organic acids needs the participation of dehydrogenase (Hanif et al., 2015; Rasul et al., 2019). Previous studies have reported that P-cycling-related genes of *phoD*, *bpp*, *gcd*, and *pstS* can encode alkaline phosphatase, phytase, glucose dehydrogenase, and phosphatase inorganic transporter system, respectively (Neal et al., 2017; Wan et al., 2020a). Therefore, *phoD*, *bpp*, *gcd*, and *pstS* genes can be good biomarkers to provide insight into soil P transformation.

Prior studies have reported that specific bacterial community including alkaline phosphomonoesterase-harboring bacterial community and phytase-producing bacterial community can promote plant growth (Maougal et al., 2014; Hanif et al., 2015; Ye et al., 2017; Wei et al., 2019). Additionally, many PSBs have been isolated from natural conditions and found to possess plant growth-promoting capability, such as *Acinetobacter* (Collavino et al., 2010; Liu et al., 2014), *Pseudomonas* (Yu et al., 2011), *Burkholderia* (Collavino et al., 2010), and *Bacillus* (Hanif et al., 2015; Hansen et al., 2020). The application of PSB in agriculture is a useful approach to enhance soil P availability and avoid excessive use of P fertilizer. Therefore, it is necessary to reveal plant growth-promoting mechanism of PSB. P solubilization and mineralization of single PSB are gradually clarified; however, effects of PSB on transformation of both inorganic and organic P and rhizosphere bacterial community are poorly understood.

To broaden candidates of P-solubilizing microorganisms, we isolated a PSB *Acinetobacter pittii* gp-1 from agricultural soils (Wan et al., 2020b). In a prior study, we found the strain *A. pittii* gp-1 showed good performances for utilizing tricalcium phosphate (TCP), aluminum phosphate, iron phosphate, and phytate (Wan et al., 2020b). Soil-derived *Acinetobacter* bacteria present good P-solubilizing abilities and show great potentials in agroecosystems (Collavino et al., 2010; Yu et al., 2011; Marra et al., 2012; Rasul et al., 2019). However, responses of diversity, composition, and function of indigenous bacterial community to inoculation of PSB *Acinetobacter* remain unknown. Soybeans are in great demand by human society, and P deficiency leads to poor growth and low production of soybean (Bononi et al., 2020). This situation caught our interest to investigate the growth-promoting capacity of *Acinetobacter* bacteria for soybean. In the present study, we aimed to (i) investigate effects of PSB inoculation on P transformation and plant growth-promoting performance and (ii) explore responses of soybean rhizosphere bacterial community to the inoculation of PSB. We hypothesized that the inoculation of PSB *A. pittii* gp-1 would increase P availability and promote the growth of plant and might elevate the P-cycling-related gene abundance. To meet our purpose and address our hypotheses, we conducted potted experiments and Illumina MiSeq sequencing and evaluated soil properties.

## Materials and Methods

### Potted Experiment Design

The previously isolated PSB *A. pittii* gp-1 (accession number: MK641660) with indole acetic acid production ability was used in potted experiment. The strain gp-1 was inoculated to 200 ml of the National Botanical Research Institute’s phosphate (NBRIP) medium and incubated at 28°C with shaking of 180 rpm for 5 days. NBRIP medium contained 10 g/L of glucose, 5 g/L of Ca_3_(PO_4_)_2_, 0.25 g/L of MgSO_4_⋅7H_2_O, 5 g/L of MgCl_2_⋅7H_2_O, 0.2 g/L of KCl, 0.1 g/L of (NH_4_)_2_SO_4_, and 2 ml/L of trace element solution (EDTA, 10 g/L; MnSO_4_⋅H_2_O, 2.2 g/L; FeSO_4_⋅7H_2_O, 1.0 g/L; CuSO_4_⋅5H_2_O, 0.5 g/L; CoCl_2_⋅6H_2_O, 0.3 g/L; Na_2_MoO_4_⋅2H_2_O, 0.2 g/L; and CaCl_2_, 0.1 g/L) (Nautiyal, 1999). After incubation, bacteria were collected by centrifuging and washed three times with sterile water.

The experimental potted soil was collected from an uncultivated field in Wuhan, China (30°28′N, 114°21′E). The soil type is calcareous, with original pH, total carbon, total nitrogen, availability phosphorus, and total phosphorus of 6.9, 0.52, 0.68%, 0.22 mg/g, and 0.89 mg/g, respectively. These P-deficient soils were sieved through a 2-mm mesh to remove stones and plant residuals. TCP was applied as phosphorus source in plant growth promotion experiment as described in previous literatures (Yu et al., 2011; Liu et al., 2014). Four potted treatments were designed: 200 g of sieved soil + 100 ml of sterile water (CK treatment), 195 g of sieved soil + 5 g of TCP + 100 ml of sterile water (Tri treatment), 200 g of sieved soil + 10 ml of bacterial suspension (10^7^ cfu/ml) + 90 ml of sterile water (Sup treatment), and 195 g of sieved soil + 5 g of TCP + 10 ml of bacterial suspension (10^7^ cfu/ml) + 90 ml of sterile water (Bac treatment). Each treatment had five replications. Soybean seeds (*Glycine max* w82) were purchased from China National Seed Group, pre-cultivated in sterile nutritious soils, and allowed them grow to about 10-cm length of sprouts. Each sprout with same growth potential was transplanted to each plastic pot as described above, and the strain gp-1 was inoculated to soybean rhizosphere in Sup and Bac treatments. Each plot was covered with Nylon membrane. These pots were randomly placed in greenhouse and incubated at 25°C with the cycling treatment of 16-h light and 8-h dark for a total of 40 days.

### Determination of Phosphate-Solubilizing Bacterium Abundance and Indole Acetic Acid

Every 10 days, we used alcohol-wiped shovels and tweezers to collected about 5 g of bulk soils near soybean root from each pot. In the experiment of plate-colony counting for abundance of PSB, 1 g of freeze-dried soil was added to 10 ml of sterile water and shaken at 180 rpm for 30 min, and the mixture is allowed to stand for 10 min. Then 1 ml of soil suspension was diluted, 0.1 ml × 10^–6^ of diluent was evenly spread on NBRIP solid medium containing 0.2 g/L of cycloheximide acting as fungicide and incubated at 28°C for 5 days. After incubation, the cfu in different plates were counted. We also estimated content of indole acetic acid by using Van Urk Salkowski reagent, and the standard approach has been described previously (Biswas et al., 2018).

### Determination of Soil Physicochemical Properties, Enzyme Activity, and Vegetation Properties

After 40-day growth of soybean, we excluded pots with the best and worst soybean growth in each treatment, and then 12 pots were left. We scraped rhizosphere soils by using a brush. We measured soil physicochemical properties, including pH, total carbon, total nitrogen, and available P, based on standard methods (Wan et al., 2021a). Microbial biomass P was evaluated by chloroform fumigation extraction and was calculated as the difference between fumigated and non-fumigated subsamples and simultaneously revised for the incomplete recovery of a P spike (Roberts et al., 2013; Ragot et al., 2016).

Soil alkaline phosphatase activity and phytase activity were determined according to previous methods (Wan et al., 2020a). Phosphatase activity and phytase activity were expressed as μg pNPP produced per gram of freeze-dried soil in 1 h and μmol P produced per gram of freeze-dried soil in 1 day, respectively.

The pots in each group was kept to measure the plant height, plant fresh weight, plant dry weight, leaf number, leaf fresh weight, root length, and root fresh weight. Soybean shoots and roots were separated from plants and dried at 60°C. The clean and dried root and shoot were separately cut into small pieces and digested by concentrated H_2_SO_4_–H_2_O_2_. The digested solutions were applied for measuring the content of root P and shoot P (Fraser et al., 2017).

### DNA Extraction, Gene Quantification, Amplicon Sequencing, and Sequence Processing

Three rhizosphere soils from each group were used to extract total DNA using a DNA extraction kit (Mo Bio, Carlsbad, CA, United States) according to the manufacturer’s instruction. DNA concentrations were determined using a NanoDrop 2,000 Spectrophotometer (Thermo Fisher Scientific, Waltham, MA, United States). All extracted DNA samples were stored at –80°C.

The absolute abundances of phosphorus-cycling-related genes in soil bacteria were measured using qPCR with SYBR green mix. Primer sequences for amplifying P-cycling-related genes (i.e., *phoD*, *bpp*, *gcd*, and *pstS*) and quantitation PCR condition are summarized in Supplementary Method 1. Additionally, we used these primers to amplify *bpp*, *phoD*, *gcd*, and *pstS* from *A. pittii* gp-1.

The V3–V4 region of bacterial 16S rRNA gene was amplified using the primers 338F (5′-ACT CCT ACG GGA GGC AGC A-3′) and 806R (5′-GGA CTA CHV GGG TWT CTA AT-3′) (Mori et al., 2013). A PCR of 20 μl was performed in triplicate using a thermal cycler (ABI 9700, Thermo, United States) and conducted at the following conditions: an initial denaturation at 95°C for 3 min, 30 cycles of 95°C for 40 s, 58°C for 40 s, and 72°C for 50 s, and then a final extension at 72°C for 10 min. Sequencing was conducted on an Illumina MiSeq platform at Majorbio Bio-Pharm Technology Co., Ltd., Shanghai, China.

The raw reads were processed to gain purified sequences following the pathway of QIIME (Caporaso et al., 2010). We eliminated (1) sequences that did not exactly match barcodes and primers; (2) sequences with an average quality score < 20; (3) sequences with maximum homopolymers < 10 bp; and (4) sequences that contained ambiguous bases call. The purified sequences were clustered into operational taxonomic units (OTUs) at 97% identity against the SILVA v128 reference set.

### Statistical Analysis

Significant differences were calculated by the one-way analysis of variance with means compared using Tukey’s test in R. Venn diagram and non-metric multidimensional scaling (NMDS) plot were used to reflect bacterial community composition. Pairwise analysis of similarity (ANOSIM) was applied to quantitatively evaluate difference in bacterial community composition by using the ‘‘anosim’’ function in the ‘‘vegan’’ package of R. Permutational multivariate analysis of variance (PERMANOVA) was applied to evaluate pure effect of factors (e.g., physicochemical parameters and enzyme activity) on vegetation properties by using the ‘‘adonis’’ function in the ‘‘vegan’’ package of R. Linear discriminant analysis (LDA) effect size (LEfSe) statistical analysis was conducted on the online interface Galaxy^1^ at a significant level of *p* < 0.05 and an LDA score > 4. Functional profiling of bacterial taxa was carried out by applying the “Tax4Fun2” package in R, and the functional redundancy index for each sample was calculated based on 16S rRNA gene similarity (Wemheuer et al., 2020). Canonical analysis of principal coordinates was applied to investigate influences of components including soil physicochemical parameters, gene abundance, cell exudates (include enzyme and indole acetic acid), and relative abundances of phylum bacteria on the vegetation properties. To identify core taxa, OTUs observed in more than 50% of all samples (> 6 samples, 875 OTUs) were applied to build a co-occurrence network. The co-occurrence network was visualized using Gephi v. 0.9.2^2^ at a significant level of *p* < 0.01 and Spearman’s correlation coefficient higher than 0.67 (Wan et al., 2021b). Structural equation model was built to show relationships among vegetation properties, physicochemical properties, gene abundance, cell exudate, and bacterial community composition by using the packages of “sem” and “plspm” in R. The first principal component (PC1) value of soil physicochemical properties, P-cycling-related gene abundance, bacterial community composition, cell exudate, and vegetation properties accounting for 96.19, 85.19, 41.56, 98.99, and 96.37% of the total variances, respectively, were used as a proxy in structural equation model.

## Results

### Shifts in Phosphate-Solubilizing Bacterium Abundance and Indole Acetic Acid Content During Soybean Growth

**FIGURE 1:**
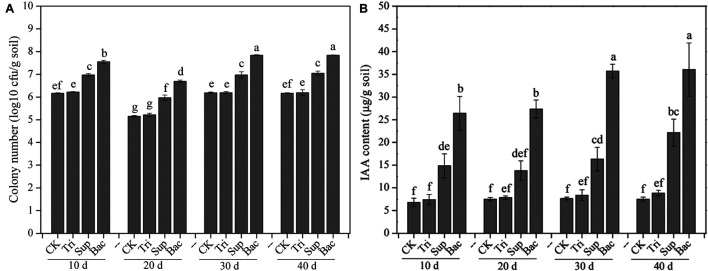
The colony-forming unit number of phosphate-solubilizing bacteria **(A)** and content of indole acetic acid **(B)** during 40 days. The results are the mean value of five replicates; error bars represent standard error. Different letters above the column indicate significance (*p* < 0.05).

The PSB abundance represented by the number of cfu showed significant difference in four treatments (CK, Tri, Sup, and Bac) during 40-day growth of soybean (Figure 1A). The abundance of PSB in Bac treatment dramatically increased from 3.57 × 10^7^ cfu/g soil at day 10 to 6.96 × 10^7^ cfu/g soil at day 40 (*p* < 0.05). The population of PSB fluctuated in CK, Tri, and Sup treatments during 40 days but did not significantly ascend at day 40 than at day 10 (*p* > 0.05). The abundance of PSB in Bac treatment was significantly higher than that in other groups; this difference might be partially due to the input of *A. pittii* gp-1 and TCP. In addition, we randomly picked 10 colonies from the plate and found that 16S rRNA gene sequence of three bacterial colonies presented 100% similarity with that of *A. pittii* gp-1.

The indole acetic acid content was significantly higher in Bac treatment than in other treatments in each period (*p* < 0.05; Figure 1B). Additionally, the indole acetic acid content noticeably increased in Bac and Sup treatments during 40 days (*p* < 0.05), while in CK and Tri treatments, it did not (*p* > 0.05). Linear regression indicated that abundance of PSB was significantly correlated with content of indole acetic acid (Supplementary Figure 1). This suggests that PSB could produce and release indole acetic acid, which in turn might promote soybean growth.

### Vegetation Properties, Soil Physicochemical Properties, and P-Cycling-Related Gene Abundance

After 40 days’ growth, the soybean presented erect leaves that became dark green (Supplementary Figure 2). Differences in vegetation properties were found in four treatments, including the plant height, plant fresh weight, plant dry weight, leaf number, leaf fresh weight, root length, root fresh weight, shoot P, and root P (Table 1). Plant dry weight, root length, shoot P, and root P were significantly higher in Bac group than in other three groups (*p* < 0.05). More importantly, the plant length, plant fresh weight, plant dry weight, leaf number, leaf fresh weight, root length, root fresh weight, root P, and shoot P were dramatically higher in Sup treatment than in CK treatment (*p* < 0.05). This suggests that the inoculation of PSB *A. pittii* gp-1 promotes soybean growth.

**TABLE 1:** Vegetation properties, soil physicochemical properties, enzyme activity, and P-cycling-related gene abundance in four potted treatments.

Property	CK treatment	Tri treatment	Sup treatment	Bac treatment
Plant height/cm	26.67 ± 6.11 (c)	42.60 ± 5.72 (bc)	62.83 ± 7.42 (ab)	88.50 ± 17.76 (a)
Plant fresh weight/g	3.12 ± 0.29 (b)	8.85 ± 0.27 (b)	26.83 ± 3.07 (a)	35.18 ± 7.14 (a)
Plant dry weight/g	0.38 ± 0.04 (c)	0.73 ± 0.25 (c)	1.53 ± 0.09 (b)	2.02 ± 0.26 (a)
Leaf number	4.67 ± 0.58 (b)	13.00 ± 1.73 (a)	14.00 ± 0.00 (a)	17.33 ± 5.77 (a)
Leaf fresh weight/g	1.09 ± 0.19 (b)	3.77 ± 0.40 (b)	10.08 ± 0.38 (a)	12.45 ± 2.78 (a)
Root length/cm	2.60 ± 0.46 (c)	8.57 ± 0.40 (b)	10.33 ± 1.15 (b)	16.33 ± 2.08 (a)
Root fresh weight/g	0.12 ± 0.02 (b)	0.58 ± 0.14 (b)	8.85 ± 1.43 (a)	12.75 ± 3.88 (a)
Shoot P/(mg/g dw plant)	5.14 ± 0.21 (c)	6.04 ± 0.46 (bc)	7.21 ± 0.29 (b)	9.27 ± 0.80 (a)
Root P/(mg/g dw plant)	1.37 ± 0.22 (c)	2.07 ± 0.24 (c)	3.57 ± 0.38 (b)	4.95 ± 0.36 (a)
Microbial P/(mg/g soil)	0.09 ± 0.01 (c)	0.12 ± 0.01 (c)	0.16 ± 0.01 (b)	0.22 ± 0.02 (a)
Available P/(mg/g soil)	0.22 ± 0.03 (c)	0.34 ± 0.06 (c)	0.63 ± 0.08 (b)	0.89 ± 0.09 (a)
pH	6.91 ± 0.15 (a)	6.76 ± 0.07 (a)	6.43 ± 0.16 (b)	6.25 ± 0.12 (b)
Total carbon (%)	0.51 ± 0.07 (c)	0.53 ± 0.04 (c)	1.40 ± 0.22 (b)	1.93 ± 0.13 (a)
Total nitrogen (%)	0.07 ± 0.01 (c)	0.08 ± 0.01 (c)	0.13 ± 0.01 (b)	0.21 ± 0.03 (a)
Phytase (μmol/g/day)	0.71 ± 0.05 (c)	0.69 ± 0.01 (c)	1.13 ± 0.11 (b)	1.53 ± 0.11 (a)
Phosphatase (μg/g/h)	4.20 ± 0.40 (c)	4.13 ± 0.14 (c)	7.76 ± 0.44 (b)	9.72 ± 0.62 (a)
*bpp* (log10 copies/g soil)	6.43 ± 0.12 (b)	6.44 ± 0.10 (b)	6.93 ± 0.21 (a)	7.20 ± 0.06 (a)
*phoD* (log10 copies/g soil)	6.36 ± 0.21 (c)	7.13 ± 0.14 (b)	7.57 ± 0.13 (a)	7.64 ± 0.05 (a)
*gcd* (log10 copies/g soil)	6.26 ± 0.13 (c)	6.67 ± 0.11 (b)	6.98 ± 0.14 (b)	7.53 ± 0.13 (a)
*pstS* (log10 copies/g soil)	7.38 ± 0.15 (b)	7.61 ± 0.06 (ab)	7.68 ± 0.47 (ab)	8.09 ± 0.18 (a)

*The results are the mean value of three replicates with standard errors. Different letters in the same row denote significance (p < 0.05).*

The soil pH (6.1–7.0) was significantly lower in Bac and Sup treatments than that in CK and Tri treatments (Table 1). Total carbon (0.43–2.04%), total nitrogen (0.07–0.24%), available P (0.19–0.99 mg/g), microbial biomass P (0.08–0.24 mg/g), alkaline phosphatase activity (3.79–10.25 μg/g/h), and phytase activity (0.66–1.63 μmol/g/day) were remarkably higher in Bac treatment than in other treatments (*p* < 0.05). These results indicate that the inoculation of PSB *A. pittii* gp-1 increases P availability and microbial activity.

Basically, the abundances of *bpp*, *phoD*, *gcd*, and *psts* genes were higher in Bac and Sup treatments than in CK and Tri treatments (Figure 1B). Expectedly, the abundance of *gcd* was significantly higher in Bac treatment (3.36 × 10^7^ copies/g soil) than that in CK treatment (1.86 × 10^6^ copies/g soil), Tri treatment (4.68 × 10^6^ copies/g soil), and Sup treatment (9.62 × 10^6^ copies/g soil). Linear regressions reflected significantly positive correlations between *phoD* gene abundance and alkaline phosphatase activity (*R*^2^ = 0.585, *p* < 0.01), between *bpp* gene abundance and phytase activity (*R*^2^ = 0.892, *p* < 0.001), and between *gcd* gene abundance and indole acetic acid content (*R*^2^ = 0.854, *p* < 0.001) (Supplementary Figure 3). Additionally, *gcd* and *pstS* could be amplified from strain *A. pittii* gp-1 using primers described above, while *bpp* and *phoD* did not. These results might imply that the addition of *A. pittii* gp-1 could increase the abundances of organic P-cycling-related bacterial abundance.

### General Properties of Rhizosphere Bacterial Community

**FIGURE 2:**
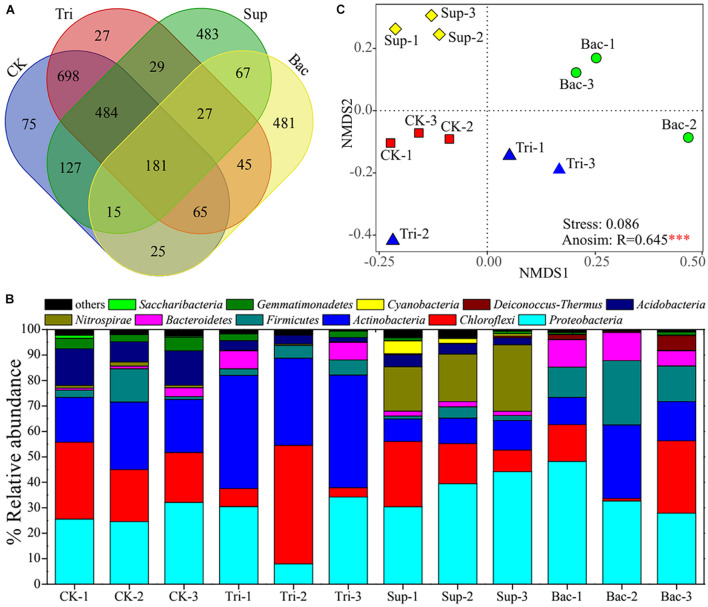
Composition of rhizosphere bacterial community. **(A)** Venn diagram shows the shared core microbiomes among four groups. **(B)** Stacking diagram reflects relative abundances of top 11 bacterial phyla (relative abundance > 1%) in 12 soil samples. **(C)** Non-metric multidimensional scaling plot exhibits difference in bacterial community composition among four treatments. Asterisks denote significance (^∗∗∗^*p* < 0.001).

A total of 2,829 OTUs were found across 12 soil samples. The CK, Tri, Sup, and Bac treatments possessed 1,670, 1,556, 1,413, and 906 OTUs, respectively; and they shared 181 OTUs (Figure 2A). A total of 39 phyla were observed, and 11 phyla with relative abundance > 0.01% were found across these 12 samples (Figure 2B). Proteobacteria, Chloroflexi, Actinobacteria, and Firmicutes were the first level dominant bacteria, with corresponding relative abundance from 7.94 to 48.22%, from 0.92 to 46.58%, from 8.86 to 44.47%, and from 1.13 to 25.25%, respectively. Acidobacteria, Bacteroidetes, Cyanobacteria, Deinococcus–Thermus, Gemmatimonadetes, Nitrospirae, and Saccharibacteria were the secondary dominant bacteria. The NMDS result showed that distinct difference in bacterial community composition among four treatments (Figure 2C). ANOSIM confirmed further the significant difference (*R* = 0.6451, *p* < 0.001). According to LEfSe result, bacteria including *Bacillus* and *Acinetobacter* were dramatically abundant in Bac treatment, while bacteria including *Acinetobacter*, *Nitrospira*, and *Rhodobacter* were significantly abundant in Sup treatment (Supplementary Figure 4). According to PERMANOVA results, the application of TCP explained 23.40% of the total variation in community composition (*F* = 5.27, *p* < 0.01), and the application of *A. pittii* gp-1 explained 29.11% of the total variation in community composition (*F* = 5.75, *p* < 0.001).

The bacterial community diversity represented by the Shannon–Wiener index (3.85–6.16) and community richness represented by Chao1 index (687–1303) were significantly lower in Bac treatment than in other treatments (*p* < 0.05; Supplementary Figure 5). This suggests that the addition of *A. pittii* gp-1 and TCP decreased rhizosphere bacterial diversity.

**FIGURE 3:**
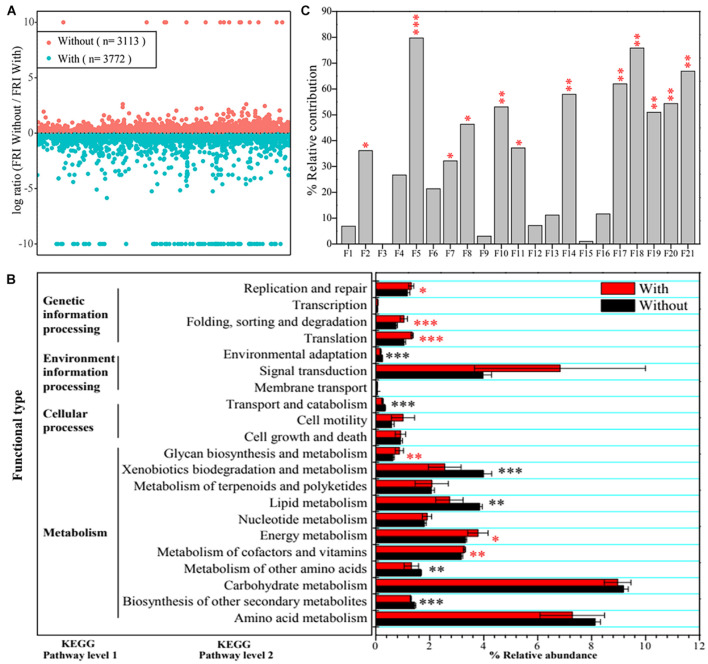
Community functional differences and functional contributions to vegetation property. **(A)** Functional redundancy indices (FRIs) of bacterial community in soils with inoculation of phosphate-solubilizing bacteria (PSBs) (Sup + Bac) and soils without addition of PSBs (CK + Tri) soils. A log ratio > 0 denotes that a function is more redundant in soils without PSB addition. **(B)** Differences in bacterial functions between group with addition of PSBs (Sup + Bac) and group without addition of phosphorus-solubilizing bacteria (CK + Tri) at Kyoto Encyclopedia of Genes and Genomes (KEGG) pathway level 1 and level 2. **(C)** Effects of functions at KEGG pathway level 2 on vegetation property determined by permutational multivariate analysis of variance (PERMANOVA). The abbreviations of F1–F21 represent functions in panel B (from bottom to up, namely, from amino acid metabolism to replication and repair). Asterisks denote significance (^∗^*p* < 0.05; ^∗∗^*p* < 0.01; ^∗∗∗^*p* < 0.001).

Based on functional profiling results, 3,113 functions at Kyoto Encyclopedia of Genes and Genomes (KEGG) pathway level 3, including carbon-, nitrogen-, phosphorus-, and sulfate-cycling-related enzymes or proteins, displayed a higher functional redundancy in CK + Tri (without *A. pittii* gp-1 addition), whereas 3,772 functions had higher redundancies in Sup + Bac (with *A. pittii* gp-1 addition) (Figure 3A). It was worth noting that 206 functions representing P-cycling-related enzymes or proteins were higher in Sup + Bac than in CK + Tri, such as phosphoglycerate dehydrogenase (EC: 1.1.1.95) and phosphoglycerate kinase (EC: 2.7.2.3). Additionally, 35 functions [(e.g., L-iduronidase (EC: 3.2.1.76), dCTP deaminase (EC: 3.5.4.30), and phloroglucinol synthase (EC: 2.3.1.253)] were unique in CK + Tri, while 198 functions [e.g., phosphotransferase (EC: 2.7.1.-), neamine phosphoribosyltransferase (EC: 2.4.2.49), 5-phosphoribostamycin phosphatase (EC: 3.1.3.88), and uracil phosphatase (EC: 3.1.3.104)] were exclusive in Sup + Bac. At KEGG level 2, some functions (e.g., metabolism of cofactors and vitamins, energy metabolism, and translation) were significantly higher in Sup + Bac than in CK + Tri (*p* < 0.05), but some functions were not (Figure 3B).

**FIGURE 4:**
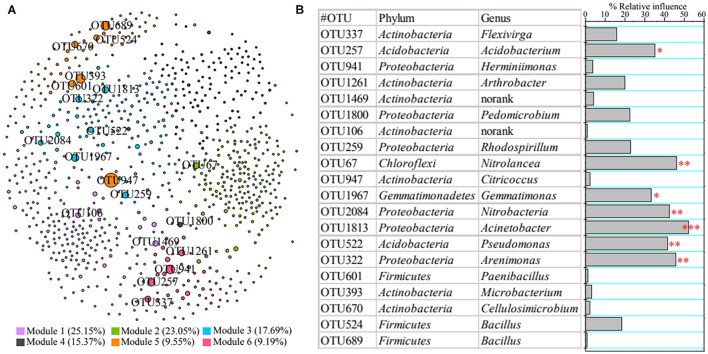
Co-occurrence network of rhizosphere bacteria **(A)** and contributions of core taxa to vegetation property based on permutational multivariate analysis of variance (PERMANOVA) **(B)**. Asterisks denote significance (^∗^*p* < 0.05; ^∗∗^*p* < 0.01; ^∗∗∗^*p* < 0.001).

A co-occurrence network was constructed to reveal the relationships among bacterial taxa (Figure 4A). We found 50,510 positive edges (represent significantly positive correlation) and two negative edges (denote dramatically negative correlation), suggesting that rhizosphere bacteria presented a less conflicting interaction. We also clarified the top 20 core nodes; i.e., those with the highest betweenness centrality were affiliated with Acidobacteria (e.g., OTU522), Actinobacteria (e.g., OTU947), Chloroflexi (OTU67), Firmicutes (e.g., OTU601), Gemmatimonadetes (OTU1967), and Proteobacteria (e.g., OTU1813) (Figure 4B).

### Effects of Abiotic and Biotic Factors on Vegetation Properties

**FIGURE 5:**
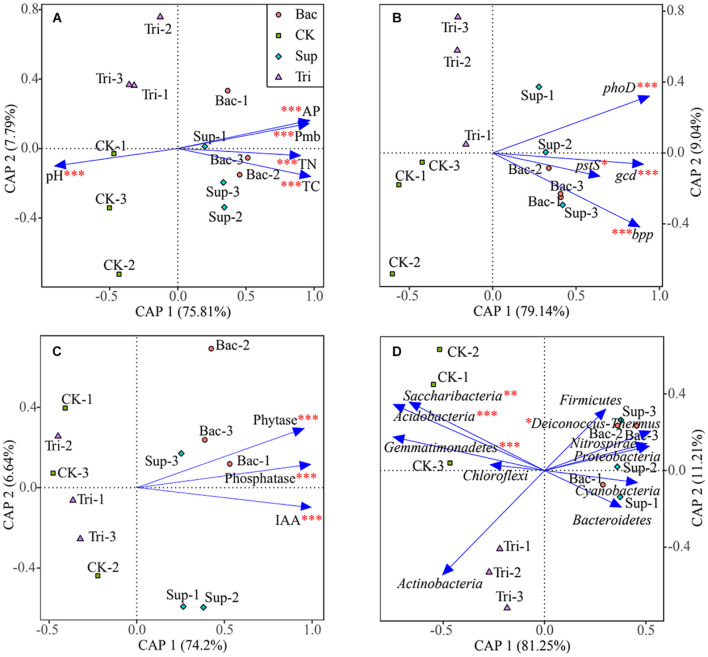
Canonical analysis of principal coordinates showing effects of soil physicochemical properties **(A)**, abundances of phosphorus-cycling-related genes **(B)**, cell exudates **(C)**, and bacterial abundances at phylum level **(D)** on vegetation properties. The significance of factors was determined using permutational multivariate analysis of variance (PERMANOVA) and is reflected by asterisks next to the variable names. Asterisks denote significance (^∗^*p* < 0.05; ^∗∗^*p* < 0.01; ^∗∗∗^*p* < 0.001).

According to PERMANOVA results, the application of TCP could explain 13.69% of the total variation (*F* = 20.23, *p* < 0.01) in vegetation properties, while the application of *A. pittii* gp-1 could explain 72.41% of the total variation (*F* = 107.05, *p* < 0.001). According to results of canonical analysis of principal coordinates, soil physicochemical properties (Figure 5A), gene abundance (Figure 5B), cell exudates (Figure 5C), and relative abundances of bacterial phyla (Figure 5D) explained more than 80% of the total variation in vegetation properties. Physicochemical parameter, gene abundance, enzyme activity and IAA, and relative abundance of bacterial phylum showed significantly pure effects on vegetation properties based on PERMANOVA (Figure 5).

Additionally, we also found that bacterial functions based on functional profiling were responsible for vegetation properties (Figure 3C). The function of metabolism of cofactors and vitamins (*R*^2^ = 79.75%, *F* = 39.38; *p* < 0.01) showed greater effect on vegetation properties than other functions according to PERMANOVA results. The core taxa identified from co-occurrence network also have significant effects on vegetation properties based on PERMANOVA (Figure 4B). The OTU1813 regarded as *Acinetobacter* genus presented higher influence (*R*^2^ = 52.08%, *F* = 10.87; *p* < 0.01) than other core taxa.

**FIGURE 6:**
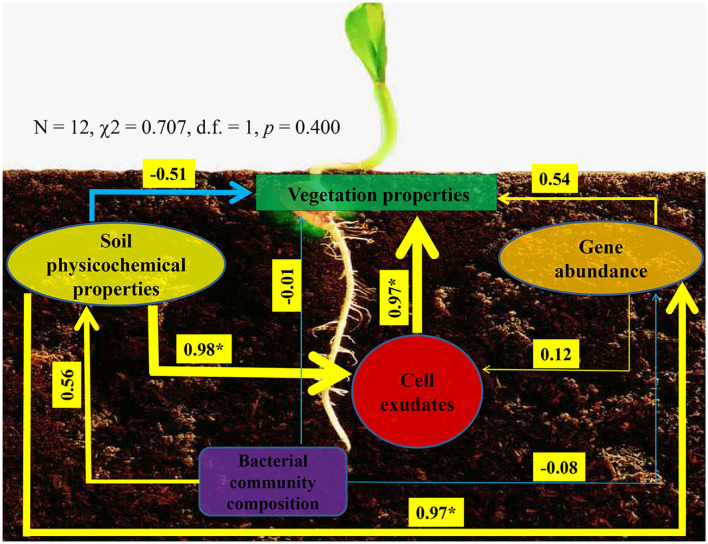
Structural equation model showing the hypothesized causal relationships among vegetation properties, soil physicochemical properties, phosphorus-cycling-related gene abundance, cell exudates (include enzyme and indole acetic acid), and bacterial community composition. The width of the arrows presents the strength of the standardized path coefficient. The blue lines indicate negative path coefficients, and yellow lines reflect positive path coefficients. Values above the lines indicate path coefficients between two parameters. Asterisks denote significance (*p* < 0.05).

Ultimately, we used structural equation model to reveal interconnections among soil physicochemical properties, P-cycling-related gene abundance, bacterial community composition, enzyme activity, and vegetation properties (Figure 6). The model presented a good fit to our data, as indicated by the non-significant χ^2^-test (*N* = 12, χ^2^ = 0.707, d.f. = 1, *p* = 0.400). On the one hand, bacterial community could affect soil physicochemical properties and P-cycling-related gene abundance, which in turn affect vegetation properties; on the other hand, soil physicochemical properties and P-cycling-related gene abundance could influence enzyme activity, which in turn influences vegetation properties. These results indicated that soil, plant, and bacteria presented close relationships.

## Discussion

Promoting efficient utilization of P is important in agriculture due to rapidly increasing cost of fertilizers and big concerns of environmental protection (Hu et al., 2018). The bacteria possessing P utilization capacity are widespread in the rhizosphere soils of different crops (Maougal et al., 2014; Hanif et al., 2015; Wan et al., 2020b) and promise great application potentials in agriculture because PSB are responsible for P availability and facilitate P uptake by crops (Richardson et al., 2011; Bononi et al., 2020; Pastore et al., 2020). However, the activity and abundance of PSB are subjected to the fertilization treatment and phosphorus fractions (Luo et al., 2017; Hu et al., 2018; Wei et al., 2019; Wan et al., 2020a). Therefore, the isolation and application of highly efficient PSB are meaningful in terms of promoting soil P availability in agroecosystems.

### Elucidating Soybean Growth-Promoting by Phosphate-Solubilizing Bacteria Acinetobacter *Pittii* gp-1

Applying PSB can increase soil available P content (Maougal et al., 2014) and promote vegetation growth (Yu et al., 2011; Biswas et al., 2018; Hansen et al., 2020). However, it should be considered whether the PSB could maintain their activity, function, and abundance after inoculation. In this study, the inoculation of PSB *A. pittii* gp-1 significantly promoted the growth of soybean represented by better vegetation properties, which is in accordance with prior findings describing that PSB can enhance the growth of legume plant (Collavino et al., 2010; Bononi et al., 2020; Cumpa-Velásquez et al., 2021) and other kinds of plants (Yu et al., 2011; Liu et al., 2014). In these studies, the increase in the content of available P or small molecular organic acid is closely correlated with the growth of plants. The PSB *Acinetobacter* genus is reported to have the ability to release small molecular organic acid (e.g., indole acetic acid, gluconic acid, oxalic acid, and citric acid) (Marra et al., 2012; Marwa et al., 2019; Rasul et al., 2019). Interestingly, we found the *A. pittii* gp-1 could produce indole acetic acid detected by using the Van Urk Salkowski reagent. Therefore, the inoculation of the *A. pittii* gp-1 might increase the content of soil organic acid, which in turn increased the content of available P. Additionally, we detected *Acinetobacter* genus in Bac treatment by using simple 16S rRNA gene sequencing for single colony. Illumina MiSeq sequencing result also reflected that *Acinetobacter* dominated in Sup and Bac groups. These results suggest that *A. pittii* gp-1 could survive after inoculation and could promote the growth of soybean.

In addition, we used four pairs of primers as described above to amplify *bpp*, *phoD*, *gcd*, and *pstS* genes from *A. pittii* gp-1. Unexpectedly, only *gcd* and *pstS* genes could be amplified. Previous studies have reported that *Acinetobacter* genus harbors *gcd* and *pstS* gene (Marra et al., 2012; Farrugia et al., 2015; Wan et al., 2020b), and almost no study has reported that *Acinetobacter* genus possesses *bpp* and *phoD* genes. However, the abundances of P-cycling-related genes including *bpp*, *phoD*, *gcd*, and *pstS* were higher in Sup and Bac treatments. These results and findings suggest that the inoculation of PSB *A. pittii* gp-1 might significantly increase both inorganic and organic P-cycling-related gene abundance of soil indigenous bacteria. This phenomenon might be due to the solubilization of inorganic P by added PSB *A. pittii* gp-1 via releasing small molecular organic acid. Consequently, part of soluble P was assimilated by native *bpp*-harboring bacteria and *phoD*-harboring bacteria and in turn enriched the abundances of *bpp* and *phoD* genes and released more phosphatase and phytase. In addition, a part of inoculated *A. pittii* gp-1 might die; thus, the cell residues could be treated as nutrient for indigenous microorganisms. Previous literatures have reported that *gcd*-harboring bacteria can produce and release small organic acid to solubilize insoluble inorganic P, thus promoting the growth of plant (Wagh et al., 2014; Rasul et al., 2019). The *bpp*-harboring bacteria and *phoD*-harboring bacteria are reported to be responsible for the turnover of soil organic P by releasing extracellular enzyme, which in return promotes the growth of vegetation (Maougal et al., 2014; Ragot et al., 2016; Hu et al., 2018; Zhang et al., 2021). Therefore, the application of PSB *A. pittii* gp-1 could enhance utilization potentials of both inorganic and organic P.

### Response of Rhizosphere Bacterial Community to Inoculation of Strain gp-1

Considering community diversity is closely correlated with soil ecosystem functions (Wan et al., 2021c), it is important to decipher effects of the application of PSB on plant rhizosphere bacterial community. We found significant decrease in rhizosphere bacterial diversity and distinct change in bacterial community composition, which is similar to findings in published literatures (Estrada-Bonilla et al., 2017; Wei et al., 2017; Widdig et al., 2019). In addition, earlier studies have reported that vegetation also affects the composition of bacterial community (Xue et al., 2017; Yang et al., 2018; Campos-Herrera et al., 2019). To the best of our knowledge, this is the first report that the addition of PSB *A. pittii* could promote the community function of rhizosphere bacteria especially phosphorus-cycling-related functions. This phenomenon might be due to elevated nutrient caused by inoculation of PSB *A. pittii*, which in turn affected rhizosphere bacterial community composition and function. An earlier study has reported that dead bacteria can be treated as available nutrient to affect growth of other microorganisms (Hanajima et al., 2019). Additionally, microbial biomass P contributes to P solubility in riparian vegetated buffer strip soils (Roberts et al., 2013).

Based on these results and findings, we raised one question of whether there were close relationships among plant, soil, and rhizosphere. The structural equation model reflected stronger interconnections among vegetation properties, soil physicochemical properties, P-cycling-related gene abundance, cell exudates, and bacterial community composition. This result is similar to our prior finding (Wan et al., 2021a). The co-occurrence network also showed that core taxa belonging to Acidobacteria, Chloroflexi, Gemmatimonadetes, and Proteobacteria presented significant effects on vegetation properties. Previous literature has reported that some specific phylum bacteria, such as Acidobacteria, Actinobacteria, and Proteobacteria, are responsible for vegetation growth under different P conditions (Bergkemper et al., 2016). Vegetation properties and microbes could also affect each other (Neal et al., 2017; Yang et al., 2018; Muñoz et al., 2021), suggesting that soil, plant, and bacteria have close relationships. In the future, we will explore molecular mechanisms to reveal close interconnections among soil, plant, and bacteria.

## Conclusion

The application of TCP and *A. pittii* gp-1 could significantly increase soil available P, enrich both inorganic and organic P-cycling-related gene abundance, and promote the growth of soybean. Addition of TCP and *A. pittii* gp-1 significantly alters the local bacterial community composition after 40-day soybean growth. To our knowledge, we firstly report that the addition of *Acinetobacter* could promote both inorganic and organic P utilization and could increase the function of rhizosphere bacterial community. Phosphate-solubilizing bacterium *A. pittii* gp-1 could be a good candidate for the growth promotion of soybean in agroecosystems, and experiments will be conducted to estimate its growth-promoting performance for more different plants in future studies.

## Data Availability Statement

The datasets presented in this study can be found in online repositories. The names of the repository/repositories and accession number(s) can be found below: https://www.ncbi.nlm.nih.gov/, SRR8742689–SRR8742700.

## Author Contributions

WW and DH designed the whole experiments. WW conducted all the experiments, analyzed the data, and wrote the manuscript. DH revised the manuscript. Both authors contributed to the article and approved the submitted version.

## Conflict of Interest

The authors declare that the research was conducted in the absence of any commercial or financial relationships that could be construed as a potential conflict of interest.

## Publisher’s Note

All claims expressed in this article are solely those of the authors and do not necessarily represent those of their affiliated organizations, or those of the publisher, the editors and the reviewers. Any product that may be evaluated in this article, or claim that may be made by its manufacturer, is not guaranteed or endorsed by the publisher.

## Footnotes

1
http://huttenhower.sph.harvard.edu/lefse/

2
https://gephi.org/
