# Supplementary material for: Phosphate-Solubilizing Bacterium Acinetobacter pittii gp-1 Affects Rhizosphere Bacterial Community to Alleviate Soil Phosphorus Limitation for Growth of Soybean (Glycine max)
Source: Front Microbiol. 2021 Sep 24;12:737116. doi: 10.3389/fmicb.2021.737116 (PMC8498572; doi:10.3389/fmicb.2021.737116)
Supplement: Supplementary file 1 [file Data_Sheet_1.docx]

Supplementary material for:

**Phosphorus-solubilizing bacterium *Acinetobacter pittii* gp-1 affects rhizosphere bacterial community to alleviate soil phosphorus limitation for growth of soybean (*Glycine max*)**

Donglan He^1^, Wenjie Wan^2, 3, 4 *^

^1^College of Life Science, South-Central University for Nationalities, Wuhan 430070, PR China

^2^Key Laboratory of Aquatic Botany and Watershed Ecology Wuhan Botanical Garden, Chinese Academy of Sciences, Wuhan 430074, PR China

^3^ Center of the Plant Ecology, Core Botanical Gardens, Chinese Academy of Sciences, Wuhan 430074, PR China

^4^State Key Laboratory of Agricultural Microbiology, Huazhong Agricultural University, Wuhan 430070, PR China

*Corresponding Author

**E-mail**: [wanwenjie@wbgcas.cn](mailto:wanwenjie@wbgcas.cn) (Wenjie Wan)

**Address**: Wuhan Botanical Garden, Lumo Road No. 1, Wuchang District, Wuhan, PR China

**Supplementary material for methods:**

**Experiment 1: Amplification for phosphorus-cycling-related genes**

Primer ALPS-F730 (5’–CAG TGG GAC GAC CAC GAG GT–3’) and primer ALPS-R1101 (5’–GAG GCC GAT CGG CAT GTC G–3’) were used to amplify *phoD* gene (Sakurai et al. 2008); Primer bppF (5’–GAC GCA GCC GAY GAY CCN GCN NTN TGG–3’) and primer bppR (5’–CAG GSC GCA NRT CAN CRT TRT T–3’) were employed to amplify *bpp* gene (Huang et al. 2009); Primer gcdF (5’–CGG CGT CAT CCG GGS NTN YRA YRT–3’) and primer gcdR (5’–GGG CAT GTC CAT GTC CCA NAD RTC RTG–3’) were applied to amplify *gcd* gene (Cleton-Jansen et al. 1990); Primer pstSF (5’–TCT ACC TGG GGA AGA TCA CAA ART GGR A–3’) and primer pstSR (5’–TGC CGA CGG GCC ANT YNW C–3’) were selected to amplify *pstS* gene (Hsieh and Wanner 2010).

Standard curves were generated using a serial dilution of a known amount of linearized recombinant plasmid containing specific gene fragment. Quantitation was performed on three technical replicates with an ABI VIIA 7 Cycle Real-time PCR System (Applied Biosystems, Foster City, CA, USA) in a 10 μL reaction system, and was conducted at 95 °C for 5 min, followed by 40 cycles of 95 °C for 15 s and 55 °C for 1 min. The amplification efficiencies for these P-cycling-related genes were as follows: *phoD*: 101%, *gcd*: 102%, *bpp*: 99%, and *pstS*: 97%. The abundances of these genes in all samples were expressed as copies per gram of freeze-dried soil.


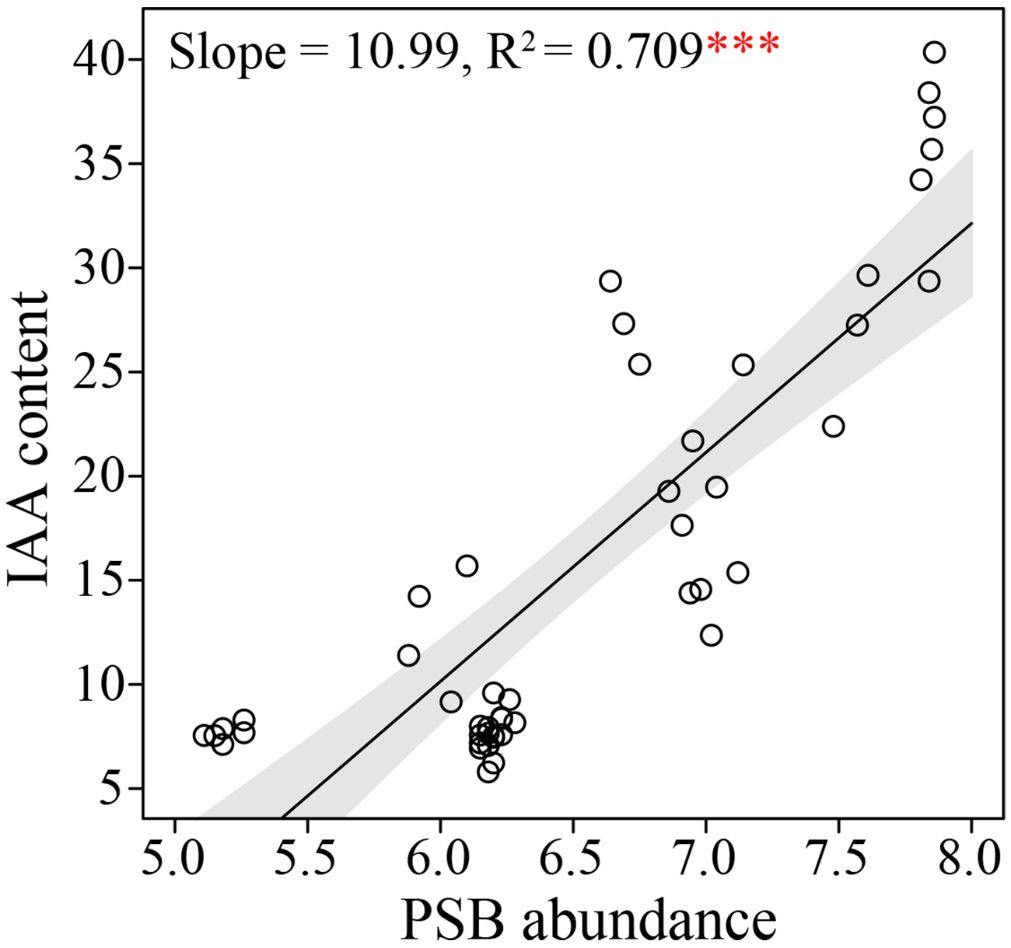


**Figure S1** Linear regression between abundance of phosphorus-solubilizing bacteria (PSB) and content of indole acetic acid. Asterisks denote significance (***, *p* < 0.001).


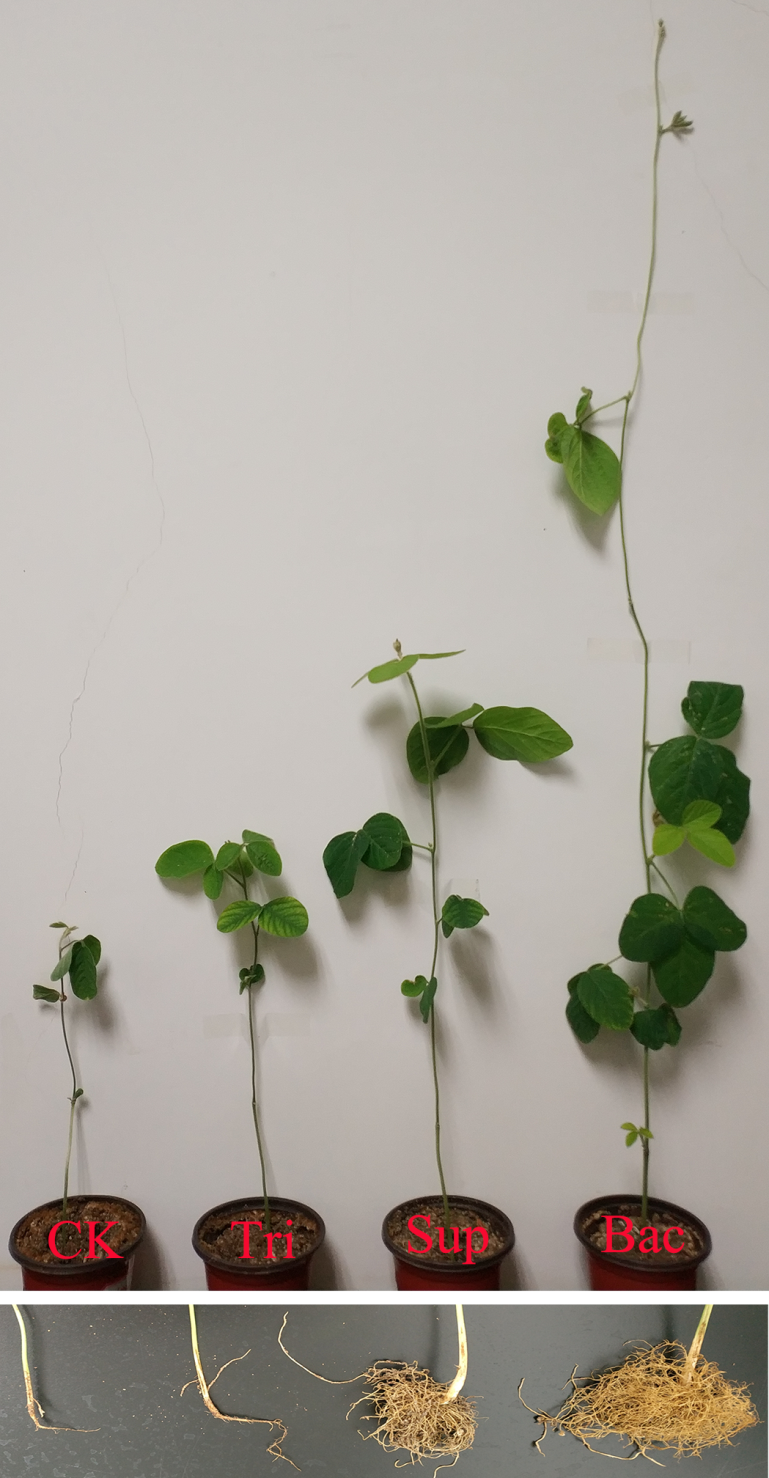


**Figure S2** Growth condition of soybean in four experimental groups after 40 days.


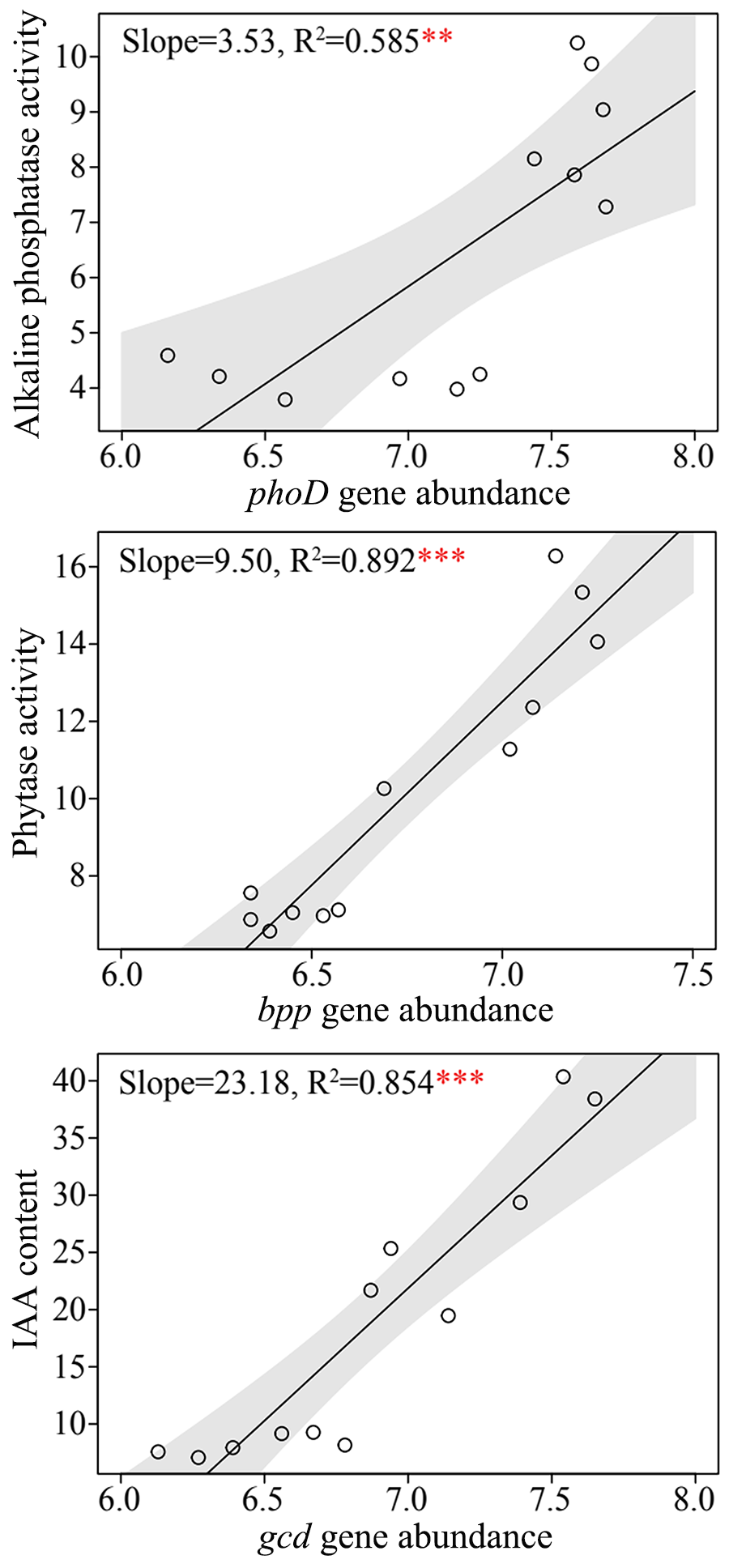


**Figure S3** Linear regression between gene abundance and cell exudate. Asterisks denote significance (***, *p* < 0.001).


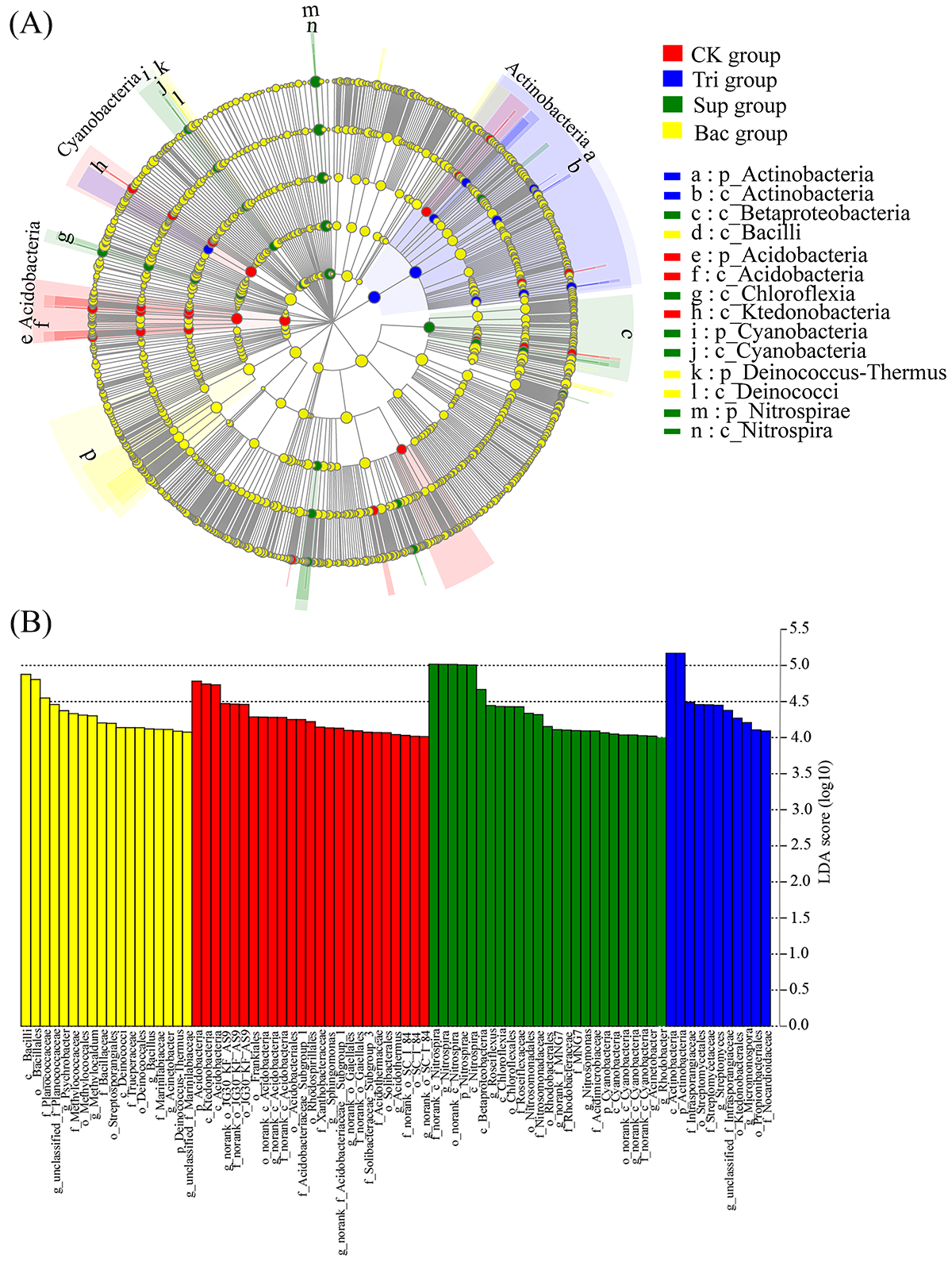


**Figure S4** Strict version of LEfSe results on bacterial community. (A) The cladogram indicates the taxa (highlighted with small circles and shading) showing different abundance values in 4 potted treatments. (B) Histogram of the LDA scores calculated for taxa differently abundance in 4 potted treatments. The abbreviations of four treatments (CK, Tri, Sup, and Bac) are defined in “Materials and methods” section.


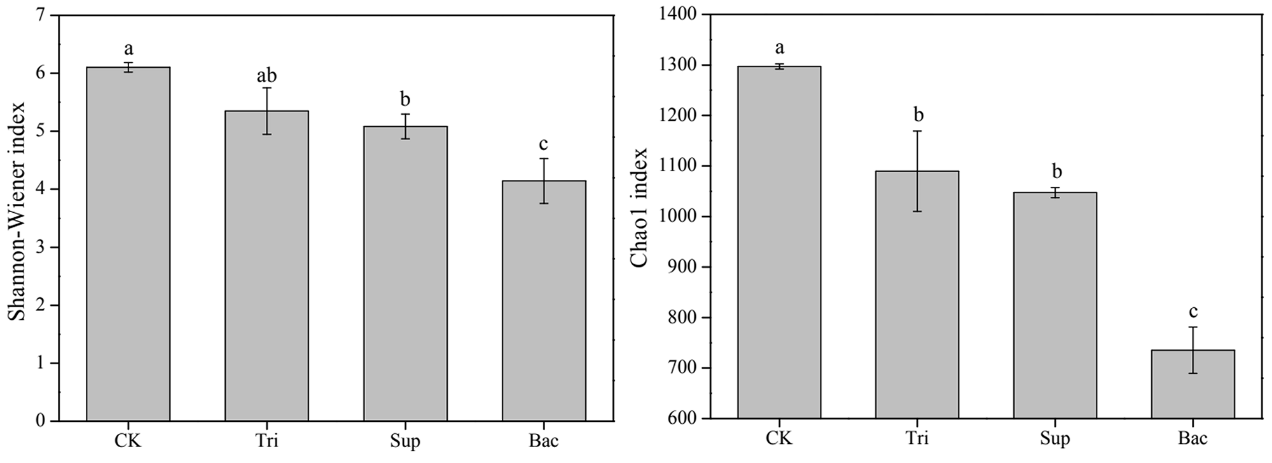


**Figure S5** Differences in Shannon-Wiener index and Chao1 index among four treatments. The abbreviations of four treatments (CK, Tri, Sup, and Bac) are defined in “Materials and methods” section. Different letters above column denote significance (*p* < 0.05).

**Reference**

Cleton-Jansen AM, Goosen N, Fayet O, van de Putte P (1990) Cloning, mapping, and sequencing of the gene encoding *Escherichia coli* quinoprotein glucose dehydrogenase. J Bacteriol 172:6308–6315

Hsieh YJ, Wanner BL (2010) Global regulation by the seven-component pi signaling system. Curr Opin Microbiol 13:198–203

Huang H, Shi P, Wang Y, Luo H, Shao N, Wang G, Yang P, Yao B (2009) Diversity of beta-propeller phytase genes in the intestinal content of grass crap provides insight into the release of major phosphorus from phytate in nature. Appl Environ Microb 75:1508–1516

Sakurai M, Wasaki J, Tomizawa Y, Shinano T, Osaki M (2008) Analysis of bacterial communities on alkaline phosphatase genes in soil supplied with organic matter. Soil Sci Plant Nutr 54:62–71
